# Immune Reaction to Type XVII Collagen Induces Intramolecular and Intermolecular Epitope Spreading in Experimental Bullous Pemphigoid Models

**DOI:** 10.3389/fimmu.2019.01410

**Published:** 2019-06-19

**Authors:** Hideyuki Ujiie, Norihiro Yoshimoto, Ken Natsuga, Ken Muramatsu, Hiroaki Iwata, Wataru Nishie, Hiroshi Shimizu

**Affiliations:** Department of Dermatology, Hokkaido University Graduate School of Medicine, Sapporo, Japan

**Keywords:** BP180, COL17, BP230, active mouse model, CD40 ligand, autoimmunity, autoantibody, NC16A domain

## Abstract

Bullous pemphigoid (BP), the most common autoimmune blistering disease, is induced by autoantibodies to type XVII collagen (COL17). Previous studies demonstrated that COL17 harbors several epitopes targeted by autoreactive T and B cells and that the target epitopes change sequentially during the disease course. To elucidate the details of the humoral immune response to COL17, we used an active BP mouse model in which BP is induced by the adoptive transfer of spleen cells from wild-type mice immunized with human COL17-expressing skin grafting to immunodeficient COL17-humanized (Rag-2^−/−^, mouse Col17^−/−^, human COL17^+^) mice. By immunoblot analysis, antibodies to the NC16A domain and other extracellular domains (ECDs) of COL17 were detected earlier than antibodies to intracellular domains (ICDs) in the active BP model. Time course analysis by enzyme-linked immunosorbent assay demonstrated a delayed peak of antibodies to ICD epitopes in active BP model. The blockade of CD40–CD40 ligand interaction soon after the adoptive transfer suppressed the production of antibodies to the non-collagenous 16A (NC16A) domain but not to an ICD epitope, suggesting the sequential activation from T and B cells against the ECD epitopes including the NC16A domain to those against ICD epitopes *in vivo*. Both wild-type mice immunized with a fragment of the NC16A domain and the recipients of those spleen cells produced IgG antibodies to ICD and ECD epitopes, showing intramolecular epitope spreading from the NC16A domain to other epitopes of COL17. Furthermore, we found that a portion of the active BP model mice show intermolecular epitope spreading from human COL17 to murine BP230. The appearance of antibodies to ICD epitopes of COL17 or of antibodies to murine BP230 did not correlate with the skin changes in the mice, suggesting that those antibodies have low pathogenicity. These results suggest that the immune response to the ECD epitopes of COL17, especially to the NC1*6A* domain, triggers intramolecular, and intermolecular epitope spreading to ICD epitopes of COL17 and to murine BP230. These novel findings provide insight into the mechanism of epitope spreading in organ-specific, antibody-mediated autoimmune disorders.

## Introduction

Bullous pemphigoid (BP) is the most common autoimmune blistering disorder and is characterized by tense blisters with itchy urticarial plaques and erythema on the entire body. BP characteristically affects the elderly, and recent studies reported a trend of increased incidence of BP (1). BP is reported to be associated with increased risk for cardiovascular disease and neurological disease (2). Autoantibodies in BP react with two structural components of the dermal-epidermal junction (DEJ): type XVII collagen (COL17, also called BP180, or BPAG2) and BP230 (also called dystonin or BPAG1). The autoantibodies to COL17 are considered to trigger the inflammatory and non-inflammatory processes, resulting in the disruption of dermal-epidermal connection. COL17 is a hemidesmosomal transmembrane protein that spans the lamina lucida and projects into the lamina densa of the DEJ (3–10). The extracellular portion of COL17 contains 15 collagenous domains separated from one another by non-collagenous domains (4). The juxtamembranous extracellular non-collagenous 16A (NC16A) domain, located at the membrane-proximal region of COL17, is preferentially recognized by autoantibodies in BP patients (11, 12). Several studies have demonstrated that the serum levels of autoantibodies to the NC16A domain of COL17 are related to the disease activity of BP (13, 14). The passive transfer of IgG antibodies to the NC16A domain of human COL17 or its murine counterpart into neonatal mice directly demonstrates the *in vivo* pathogenicity of those antibodies (15, 16). Thus, the NC16A domain of COL17 contains the major pathogenic epitope for BP. In addition, it is well-known that the intracellular domain (ICD) and the extracellular domain (ECD) of COL17 are also targeted by autoantibodies of BP (17, 18). A previous study demonstrated that 47% of BP sera reacted to the C-terminal region of COL17 (19). Autoantibodies to the C-terminal region of COL17 are thought to be involved in mucous membrane pemphigoid (7). Furthermore, a recent study demonstrated that autoantibodies in BP patients which react to the full-length recombinant COL17 protein but not to the NC16A domain preferentially react to epitopes within the mid-portion of the ECD of COL17 (20).

BP230 is another autoantigen of BP and was originally identified as the major antigen of BP (21, 22). BP230 is a cytoplasmic component of hemidesmosomes that belongs to the plakin family; it promotes the linkage of keratin intermediate filaments to hemidesmosomes (23). More than 80% of BP sera show reactivity to BP230 (24, 25). It remains uncertain whether anti-BP230 autoantibodies directly contribute to blister formation or whether they are just by-products of epitope spreading associated with disease extension, although several studies have pointed to the pathogenicity of autoantibodies to BP230 (26–28).

Epitope spreading is a phenomenon in which the targets of T- and/or B-cell responses can extend from the initial dominant epitope to other epitopes on the same protein (intramolecular epitope spreading) or to other proteins in the same tissue (intermolecular epitope spreading) over time (29, 30). Intramolecular epitope spreading has been reported in several autoimmune disorders, such as multiple sclerosis (31) and myasthenia gravis (32). It is well-known that epitope spreading frequently occurs in BP. *In vivo* experiments using a human COL17-expressing skin-grafted BP mouse model showed that IgG antibodies to human COL17 initially react to the ECD epitopes and that, subsequently, the humoral immune responses target additional ECD and ICD epitopes (33). A prospective multicenter study demonstrated that 49% of 35 BP patients showed epitope spreading that preferentially occurred at an early stage of the disease and was associated with disease severity (34). Thus, epitope spreading has been shown in both experimental murine BP and human BP. However, many questions remain, such as whether the T- and B-cell interactions for different epitopes of COL17 occur at different times and whether an immune response to the NC16A domain of COL17 actually triggers intramolecular epitope spreading to other epitopes of COL17 and/or intermolecular epitope spreading to other hemidesmosomal antigens.

To address these issues, we utilized an active disease model for BP that we previously established (35). It is generated by the adoptive transfer of human COL17-immunized spleen cells into adult immunodeficient *Rag-2*^−/−^/COL17-humanized (*COL17*^*m*−/−, *h*+^) mice. This model continuously produces IgG antibodies to human COL17 in a CD4^+^ T-cell-dependent manner and reproduces the BP disease phenotype. By using this active BP model, the current study demonstrates that the production of antibodies to the ECD epitopes of COL17 precedes that to the ICD epitopes, especially to the inner portion of the ICD. The interference of T- and B-cell interaction by monoclonal antibody to the CD40 ligand (CD40L) shows that T- and B-cell interactions for ECD epitopes precede those to ICD epitopes of COL17 in an active BP model. Wild-type mice that were immunized with a fragment of the NC16A domain produced antibodies to ICD epitopes of COL17. Furthermore, the active BP model generates antibodies to murine BP230 as a result of intermolecular epitope spreading. These findings clarify the details of epitope spreading in BP.

## Materials and Methods

### Mice

C57BL/6-background *Rag-2*^−/−^ mice were received as a gift from the Central Institute for Experimental Animals (Kawasaki, Japan). We crossed *COL17*^*m*−/−, *h*+^ (COL17-humanized) mice that we had previously generated (16) with *Rag-2*^−/−^ mice to produce *Rag-2*^−/−^*/COL17*^*m*−/−, *h*+^ (Rag-2^−/−^/COL17-humanized) mice.

### Generation of Recombinant Proteins and Synthesized Peptides

Recombinant proteins covering all parts of human COL17 were generated in a previous study (36). Briefly, human ICD-1 (amino acids Met^1^ to Ser^204^), ICD-2 (Thr^197^ to Lys^466^), NC16A (Glu^490^ to Arg^566^), ECD-1 (Gly^567^ to Gly^860^), ECD-2 (Leu^853^ to Gly^1218^), and ECD-3 (Ser^1211^ to Pro^1497^) were generated as GST-fusion proteins using the expression vector pGEX6P-1 (GE Healthcare) and the bacteria BL21 (GE Healthcare). These GST-fusion proteins were purified using GSTrap HP (GE Healthcare) according to the manufacturer's instructions. NC16A-R7 (Asp^522^ to Gln^545^), 2 ICD peptides (ICD-149: Ala^149^ to Ser^172^, ICD-320: Thr^320^ to Lys^343^) and 3 ECD peptides (ECD-917: Lys^917^ to Ser^940^, ECD-1084: Ser^1084^ to Pro^1107^, ECD-1330: Ala^1330^ to Gly^1353^) of human COL17 were chemically synthesized (Greiner Bio-One, Kremsmünster, Austria). The amino acid numbering system is based on the human COL17 sequence (NP_000494.3). Recombinant proteins covering all parts of murine BP230 were generated as previously reported (37). Briefly, total RNA of murine BP230 was extracted from murine keratinocytes, and cDNAs were synthesized by reverse transcription polymerase chain reaction. Plasmid vectors of 3 fragments of murine BP230 with His-tag at the C-terminus were designed. The 3 cDNA fragments were named BP230-1 (Met^1^-Gly^3264^), containing the N-terminal (plakin) domain; BP230-2 (Glu^3223^-Pro^5562^), containing the (coiled-coil) rod domain; and BP230-3 (Asp^5533^-Gln^7833^), containing the C-terminal (intermediate filament-binding) domain, according to GenBank (AF396877.1) and a previous report (38). These cDNAs were inserted into the pSeq Tag2/Hygro B vector (Thermo Fisher Scientific). Vectors were transfected into HEK293 cells with Lipofectamine 2000 (Thermo Fisher Scientific) for transient expression of BP230. Supernatants were collected and centrifuged for purification by using Amicon Ultra Centrifugal Filters (Merck Millipore, Darmstadt, Germany).

### Generation of the Active BP Model

To make an active BP model, we first immunized wild-type mice with human COL17-expressing mouse skin graft according to the reported method (39). Briefly, full-thickness 1-cm^2^ pieces of dorsal skin were removed from sacrificed COL17-humanized mice and grafted onto the backs of gender-matched, 6- to 8-week-old C57BL/6 wild-type mice. After the topical application of antibiotic ointment, the grafted site was covered with gauze and an elastic bandage for 14 days. Antibody production was confirmed at 5 weeks after skin grafting by immunofluorescence (IF) analysis, as described below. Spleen cells were isolated 5 weeks after the skin graft and pooled from several immunized wild-type mice, and 2.0 × 10^8^ cells/mouse were adoptively transferred into *Rag-2*^−/−^/COL17-humanized mice through a tail vain in 500 μL of PBS as previously reported (35). In some experiments, the wild-type mice were immunized at the hind footpad with 50 μg NC16A-R7 (fused with KLH) peptides emulsified in the adjuvant TiterMax Gold (TiterMax USA, Norcross, GA). The mice received an additional boost at the tail base 1 week after initial immunization. Negative controls were generated by immunization with phosphate-buffered saline (PBS) and TiterMax Gold. Spleen cells were isolated 5 weeks after immunization and pooled from several immunized wild-type mice, and 1.0 × 10^8^ cells/mouse were transferred into *Rag-2*^−/−^/COL17-humanized mice by retroorbital injection in 100 μL of PBS as previously described (40).

### *In vivo* Anti-CD40L Antibody Treatment

*Rag-2*^−/−^/COL17-humanized recipients that were adoptively transferred with immunized spleen cells were intraperitoneally injected with 1,000 μg hamster monoclonal antibody MR1 specific to mouse CD40L (Taconic Farms, Hudson, NY) at day 0 soon after the adoptive transfer of immunized splenocytes as previously reported (41). All of the treated mice were carefully observed for at least 10 weeks after adoptive transfer.

### Evaluation of Recipient Mice

Weekly, the recipient mice were examined for general condition and for percentage of body surface area affected by cutaneous lesions (i.e., erythema, hair loss, blisters, erosions, and crusts). Serum samples were also obtained from recipient mice weekly and assayed by indirect IF microscopy and enzyme-linked immunosorbent assay (ELISA).

### Indirect IF Study

Indirect IF using mice sera was performed on normal human skin (NHS) and wild-type mouse skin using standard protocols. We used 1:20 diluted mouse sera as the primary antibodies and 1:100 diluted FITC-conjugated antibodies to murine IgG (Jackson ImmunoResearch Laboratories, West Grove, PA) as the secondary antibodies.

### Immunoblot Analysis

Immunoblotting of recombinant proteins covering human COL17 was performed as described previously (36). Briefly, each sample was solubilized in Laemmli's sample buffer and applied to SDS-polyacrylamide gels and then transferred to a nitrocellulose membrane. The membrane was blocked for 1 h at room temperature in 3% skimmed milk in TBS and then incubated with 1:20 diluted mouse serum samples overnight at 4°C. Bound antibodies were visualized using 1:500 diluted HRP-conjugated goat anti-rat IgG (Jackson ImmunoResearch). Color was developed with 4-chloro-1-naphthol in the presence of H_2_O_2_. For the detection of antibodies to murine BP230, recombinant murine BP230 proteins were used as a substrate. The membranes were incubated with 1:20 diluted mouse serum samples overnight at 4°C. Bound antibodies were visualized using 1:5,000 diluted HRP-conjugated goat anti-rat IgG (Jackson ImmunoResearch). As a positive control, 1:5,000 HRP-conjugated mouse anti-His-tag mAb-HRP-DirecT (MBL, Nagoya, Japan) was used. The blots were detected using the ECL Plus Detection Kit (GE Healthcare, Fairfield, CT).

### ELISA

To determine the titer of antibodies to the NC16A domain of human COL17 in serum samples from the experimental mice, 96-well microtiter plates coated with recombinant NC16A protein purchased from MBL were incubated with diluted mouse sera for 1 h at room temperature. After being washed, bound antibodies were developed with 1:40,000-diluted, HRP-conjugated antibodies to mouse IgG (Jackson ImmunoResearch Laboratories), and the OD was read at 450 nm. The ELISA index value was defined by the following formula: index = (OD_450_ of tested serum—OD_450_ of negative control)/(OD_450_ of positive control—OD_450_ of negative control) × 100 (35). To examine the titers of antibodies to various regions of human COL17, synthesized peptides in 0.1 M sodium carbonate buffer (pH 9.5) were coated on F96 Maxisorp Nunc-Immuno plates (Thermo Scientific, Roskilde, Denmark) at 5 μg/ml and left overnight at 4°C. The plates were washed three times with PBS containing 0.05% Tween 20 and were blocked for 1 h at room temperature with ELISA assay diluent (BD Biosciences). They were incubated for 2 h at room temperature with 1:100-diluted mouse sera for NC16A-R7 or 1:25 for other peptides in assay diluent. The plates were washed four times and were incubated for 30 min at room temperature with 1:20,000-diluted HRP-conjugated antibodies to mouse IgG for NC16A-R7 or 1:10,000 for other peptides in an assay diluent. After four washings, the plates were displayed in a 1:1 mixture of substrate reagent A containing hydrogen peroxide and substrate reagent B containing tetramethylbenzidine (BD Biosciences) and were then stopped with 1 M phosphoric acid (BD Biosciences). The results were shown by OD at 450 nm. In some experiments, binding activities were calculated by the following formula: index value = (OD_450_ of tested serum—OD_450_ of negative control)/(OD_450_ of positive control—OD_450_ of negative control) × 100.

### Statistical Analyses

Data were expressed as mean ± SEM. Statistical analyses were performed using GraphPad Prism (GraphPad Software, La Jolla, California, USA). The unpaired *t*-test with Welch's correction was used for comparisons of ELISA index in wild-type mice grafted with human COL17-expressing skin (skin-grafted wild-type mice). *P*-values of <0.05 were considered significant compared with the control.

## Results

### Antibodies to the NC16A Domain of COL17 Decreased Earlier Than Those to the DEJ in the Active BP Model

**Figure 1:**
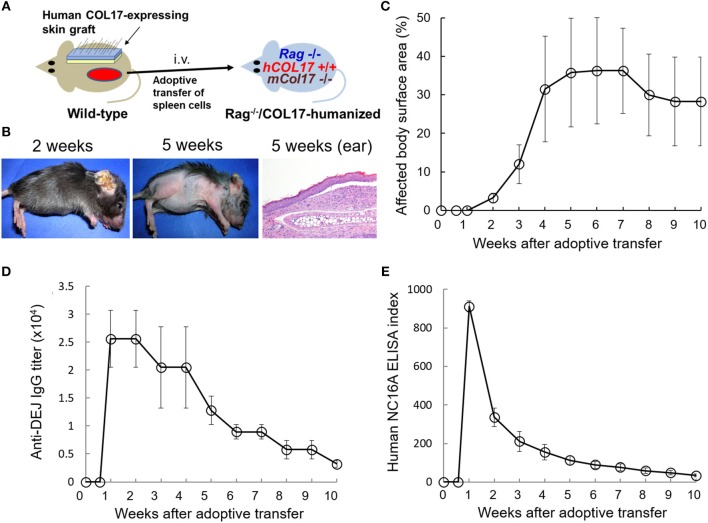
Antibodies to the NC16A domain of COL17 decrease more rapidly than those to the dermal-epidermal junction (DEJ) in an active BP mouse model. **(A)** Schematic of the generation of an active BP mouse model. **(B)** Representative clinical presentations of the active BP model at 2 and 5 weeks after the adoptive transfer of immunized spleen cells and hematoxylin and eosin staining of the ear at 5 weeks (original magnification × 20). **(C)** Time course of disease severities for the *Ra*g^−/−^/COL17-humanized recipients (*n* = 4). **(D)** Time course of titers of circulating IgG antibodies to the dermal-epidermal junction (DEJ) of the skin as determined by indirect IF using sera of an active BP model and normal human skin (*n* = 4). **(E)** Time course of the titers of circulating IgG antibodies to the NC16A domain as determined by ELISA (*n* = 4). Results are shown as mean ± SEM.

First, we prepared the active BP mouse model by the adoptive transfer of spleen cells from a wild-type mouse that was immunized with human COL17-expressing skin grafting onto adult immunodeficient *Ra*g^−/−^/COL17-humanized mice (*n* = 4) (Figure 1A). Because human COL17-expressing skin contains full-length human COL17, the skin grafting theoretically induces polyclonal IgG antibodies to various regions of human COL17 in the skin-grafted wild-type mice as well as *Ra*g^−/−^/COL17-humanized recipient mice. As we previously reported (35), around day 7 after the adoptive transfer, the *Ra*g^−/−^/COL17-humanized recipients began to scratch their snouts, ears, and chests. Patchy hair loss with erythema started to develop on the neck and chest around 14 days after the adoptive transfer in the recipients. Ear swelling with crusts was also observed (Figure 1B). The dehaired patches gradually enlarged and spread to other regions on the trunk, face and extremities over the next 2–4 weeks, resulting in large areas of alopecia (Figure 1B). Dermal-epidermal separation with mild inflammatory cell infiltration was also seen in some of the ear samples at 5 weeks (Figure 1B). The affected body surface area plateaued 6 weeks after the transfer and remained high until 10 weeks after the transfer (Figure 1C).

Indirect IF examination using sera from the active BP model revealed that IgG bound to the DEJ of normal human skin. Time-course analysis revealed that IgG antibodies to the DEJ, which reflects the presence of antibodies to human COL17, became detectable in recipients' sera 1 week after the transfer. Although the titer gradually decreased after the peak, it remained high (> × 2,560) for 10 weeks (Figure 1D). We next examined the titer of antibodies to the NC16A domain of human COL17 by ELISA (MBL, Nagoya Japan). ELISA analysis revealed that antibodies to the NC16A domain of COL17 appeared in the recipients' sera as early as 1 week after the transfer. The antibody level peaked around day 9 after the transfer and then rapidly decreased, falling to a low level (<10% of the peak) at 6 weeks after the transfer (Figure 1E). These results demonstrate that the titers of total antibodies to human COL17 remained elevated for longer than did those to the NC16A domain, suggesting the presence of additional antibodies which are elevated in the late phase of the disease.

### Antibodies to ECD and ICD Epitopes of COL17 Become Detectable at Different Times in the Active BP Model

**Figure 2:**
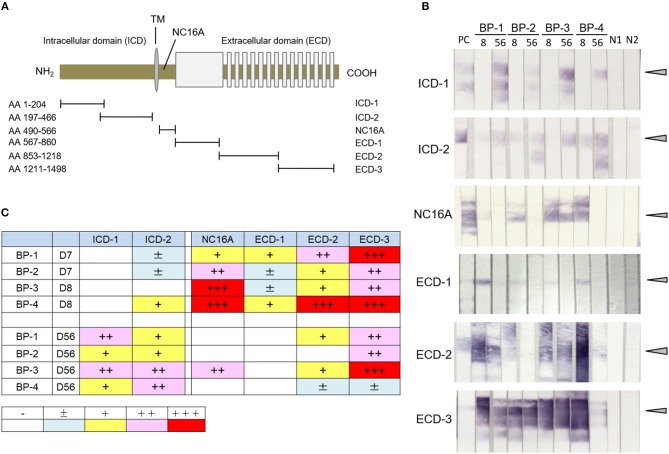
Antibodies to ICD epitopes of COL17 become detectable later than those to ECD epitopes by immunoblotting in an active BP model. **(A)** Schematic of human COL17 and recombinant proteins. TM, transmembrane domain; NH_2_, N-terminus; COOH, C-terminus; AA, amino acid. **(B)** Reactivity of sera from active BP model mice (*n* = 4, days 8 and 56) to fragments of COL17 as measured by immunoblotting. PC; positive control (BP patient serum), 8, day 8; 56, day 56; BP-1-4, active BP model mice #1-4, respectively; N1, negative control-1 (wild-type serum); N2, negative control-2 (normal human serum). Arrowheads indicate positive levels. **(C)** Relative intensities of reactivity as measured by immunoblotting. –, negative; ±, faint; +, weakly positive; ++, positive; +++, strongly positive.

To examine the presence of antibodies to various regions of human COL17 in the active BP model, we utilized 6 fragments of COL17 which were previously reported (Figure 2A) (36). By immunoblotting using those fragments from 4 active BP model mice, we analyzed the sera at days 8 and 56. All sera at day 8 strongly reacted to the NC16A domain and ECD fragments, but only one serum clearly reacted to ICD-2 and no serum bound to ICD-1 (Figure 2B). Interestingly, the reactivity of sera to NC16A, ECD-1, and ECD2 was apparently decreased at day 56, while the reactivity to ICD-2 was increased and ICD-1 was recognized by all sera (Figure 2B). The relative intensity of the reactivity is summarized in Figure 2C. To confirm these findings, we additionally analyzed sera from 4 active BP model mice at days 8, 35, and 56 after the adoptive transfer, and they showed a similar tendency (Supplementary Figure 1) Thus, antibodies to ECD epitopes of human COL17 are detectable from the early phase of the disease, whereas antibodies to ICD epitopes, especially to the inner portion of the ICD, were elevated in the late phase in the active BP model.

### The Peak of Antibodies to an ICD Epitope of COL17 Lags That to the NC16A Domain and ECD Epitopes in the Active BP Model

**Figure 3:**
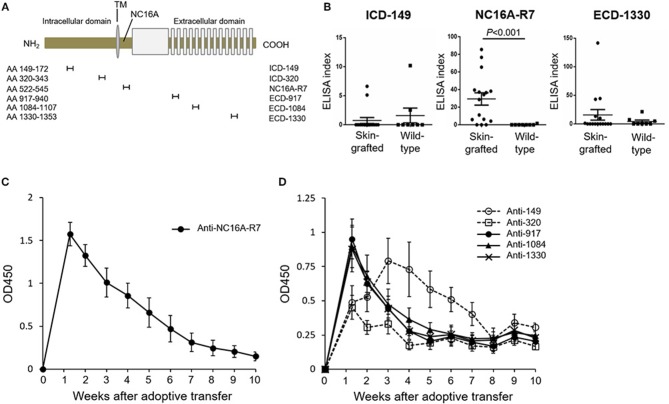
The peak of antibodies to ICD-149 lags that of antibodies to the NC16A domain and ECD epitopes by ELISA in an active BP model. **(A)** Schematic of human COL17 and synthesized peptides. TM, transmembrane domain; NH_2_, N-terminus; COOH, C-terminus; AA, amino acid. **(B)** ELISA index of skin-grafted wild-type sera (day 35, *n* = 16) and untreated wild-type sera (*n* = 8) measured by ELISA. Sera were diluted to 1:100 for NC16A-R7 and 1:25 for ICD-149 and ECD-1330. **(C)** Time course of titers of circulating IgG antibodies to NC16A-R7 as determined by ELISA (*n* = 6). **(D)** Time course of titers of circulating IgG antibodies to ICD-149, ICD-320, ECD-917, ECD-1084 and ECD-1330 as determined by ELISA (*n* = 6, respectively). Results are shown as mean ± SEM.

To investigate the time course of antibodies to various epitopes of human COL17 in the active BP model, we newly generated an ELISA using synthesized peptides of human COL17. As a fragment of the NC16A domain, we utilized the NC16A-R7 (Asp^522^ to Gln^545^) peptide because we previously reported that IgG antibodies to R7, but not to other portions of the NC16A domain, are pathogenic *in vivo* (36). We also synthesized 2 ICD peptides (ICD-149: Ala^149^ to Ser^172^, ICD-320: Thr^320^ to Lys^343^) and 3 ECD peptides (ECD-917: Lys^917^ to Ser^940^, ECD-1084: Ser^1084^ to Pro^1107^, ECD-1330: Ala^1330^ to Gly^1353^) with reference to previous studies (18, 33, 42) (Figure 3A). First, we measured the titers of antibodies in skin-grafted wild-type mice (*n* = 16) at 5 weeks after the skin grafting and compared them to those in untreated wild-type mice (*n* = 8) by ELISA. Although we expected the skin grafting to induce antibodies to every epitope of human COL17, it preferentially induced antibodies to NC16A-R7 but not to ICD-149 nor to ECD-1330 (Figure 3B). Next, we examined the titers of antibodies in the active BP model (*n* = 6). As expected, the titer of antibodies to NC16A-R7 peaked at day 9 and decreased rapidly thereafter (Figure 3C). The titers of antibodies to ECD-917, −1084 and −1330 also peaked at day 9 and then rapidly decreased, similar to the antibodies to NC16A-R7 (Figure 3D). The titer of antibodies to ICD-320 also peaked at day 9 and remained stable after the peak. Notably, the titer of antibodies to ICD-149 slowly increased and peaked at day 21 and then gradually decreased (Figure 3D). It should be noted that the titers of antibodies to NC16A-R7 were far higher than those to other epitopes of COL17, because 1:100 and 1:25 diluted sera were used to check the reactivity to NC16A-R7 and to other peptides, respectively. These results suggest that humoral immune response occurs preferentially to the NC16A domain in skin-grafted wild-type mice and then spreads to other epitopes of COL17 in the active BP model in which the response to ECD epitopes precedes that to ICD epitopes.

### Blockade of CD40–CD40 Ligand Interaction at Day 0 Preferentially Suppresses the Production of Antibodies to the NC16A Domain but not to Other Epitopes of COL17

**Figure 4:**
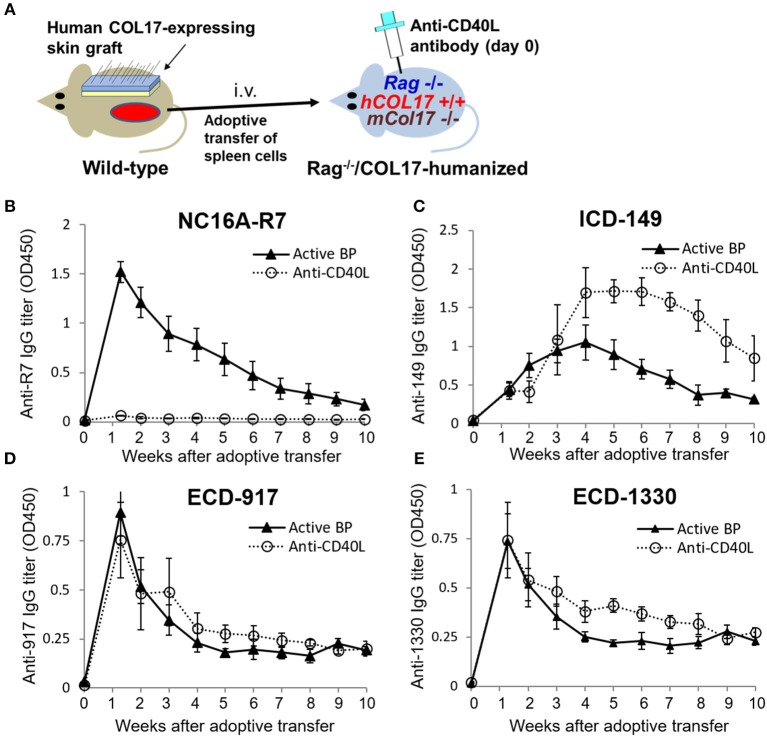
Blockade of CD40–CD40 ligand interaction preferentially decreases antibodies to the NC16A domain but not to other epitopes of COL17. **(A)** Schematic of treatment of the active BP model with antibodies to murine CD40 ligand (CD40L). **(B–E)** Time course of titers of circulating IgG antibodies to NC16A-R7 **(B)**, ICD-149 **(C)**, ECD-917 **(D)**, and ECD-1330 **(E)** in an anti-CD40L antibody-treated active BP model (*n* = 4) and in an untreated active BP model (*n* = 9) as determined by ELISA. Results are shown as mean ± SEM.

To further investigate the timing of T- and B-cell responses to various epitopes of COL17 in an active BP model, we interfered with the interaction between T and B cells by administrating monoclonal antibodies to CD40 ligand (CD40L) at day 0 of the adoptive transfer (*n* = 4) and compared the titers of antibodies in untreated active BP model (*n* = 9) (Figure 4A). As we previously reported (41), a single dose of antibodies to CD40L at day 0 strongly suppressed the production of antibodies to the NC16A domain (Figure 4B). Antibodies to ECD epitopes of COL17 (ECD-917 and 1330) were not suppressed by the treatment (Figures 4D,E). Interestingly, the peak of antibodies to ICD-149 was increased and prolonged by the treatment compared to the untreated active BP model (Figure 4C). These results suggest that T- and B-cell interaction for the ECD epitopes occurs very soon after adoptive transfer, whereas the interaction regarding ICD epitopes occurs late, so it is unaffected by the early treatment of antibodies to CD40L.

### Immunization With NC16A-R7 Induces Antibodies to ICD and ECD Epitopes of COL17 *in vivo*

**Figure 5:**
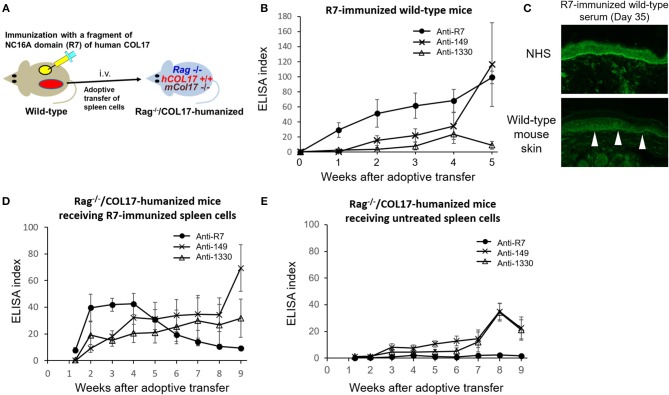
The immune response to the NC16A domain spreads to ICD and ECD epitopes of COL17 *in vivo*. **(A)** Schematic of the immunization of wild-type mice with NC16A-R7 and the adoptive transfer of spleen cells to make the active BP model. **(B)** Time course of titers of circulating IgG antibodies to NC16A-R7, ICD-149, and ECD-1330 in NC16A-R7-immunized wild-type mice (*n* = 5) as determined by ELISA. **(C)** Representative images of IgG deposition in indirect IF study using normal human skin (NHS) and wild-type mouse skin as substrates and 1:20 diluted sera from NC16A-R7-immunized wild-type mice at day 35 as primary antibodies. Arrowheads indicate IgG deposition around the basal keratinocytes. **(D,E)** Time course of titers of circulating IgG antibodies to NC16A-R7, ICD-149, and ECD-1330 in *Ra*g^−/−^/COL17-humanized mice that received spleen cells from NC16A-R7-immunized mice (*n* = 8) **(D)** and in those that received spleen cells from untreated wild-type mice (*n* = 8) **(E)** as determined by ELISA. Results are shown as mean ± SEM.

Because COL17-humanized skin contains full-length human COL17, skin grafting can activate lymphocytes to multiple epitopes of COL17 in wild-type mice. To simply analyze epitope spreading from the NC16A domain to other domains of COL17, we immunized wild-type mice with NC16A-R7 peptides fused with KLH twice at days 0 and 7 (Figure 5A). The immunized mice started to produce antibodies to NC16A-R7 within 1 week. However, interestingly, the mice also produced antibodies to ICD-149, which were detected from 2 weeks after the immunization and which gradually increased (Figure 5B). Meanwhile, the titers of antibodies to ECD-1330 were very low. Indirect IF study demonstrated that NC16A-R7-immunized wild-type sera reacted to the DEJ of normal human skin (NHS) (positive/total = 5/5). Additionally, they reacted to the surface of basal keratinocytes in wild-type mouse skin (positive/total = 4/5) (Figure 5C), suggesting the presence of antibodies that recognize murine COL17 and/or other keratinocyte antigens. We transferred the spleen cells to *Rag-2*^−/−^/COL17-humanized mice at 5 weeks after immunization (*n* = 8) (Figure 5A). As a control, spleen cells obtained from untreated wild-type mice were transferred to *Rag-2*^−/−^/COL17-humanized recipients (*n* = 8). As expected, the recipients receiving NC16A-R7-immunized spleen cells developed antibodies to NC16A-R7 shortly after adoptive transfer, and then the titer gradually decreased (Figure 5D). Additionally, antibodies to ICD-149 and ECD-1330 became detectable 2 weeks after transfer and then slowly increased. These results indicate that the re-activation of T and B cells to ICD-149 occurs later than that to NC16A-R7 in the recipients. All the recipients developed faint or no skin changes that accounted for <2% of skin surface area, suggesting that the immune response in the recipients is unrelated to the inflammation of the skin. Notably, the recipients of spleen cells from the untreated wild-type mice produced low titers of antibodies to ICD and ECD epitopes but not to the NC16A domain at a late phase (Figure 5E), suggesting that a small number of naïve T and B cells from untreated wild-type mice can weakly react to ICD and ECD epitopes of human COL17 in the recipients.

### A Portion of the Active BP Model Mice Produce a Low Titer of Antibodies to Murine BP230

**Figure 6:**
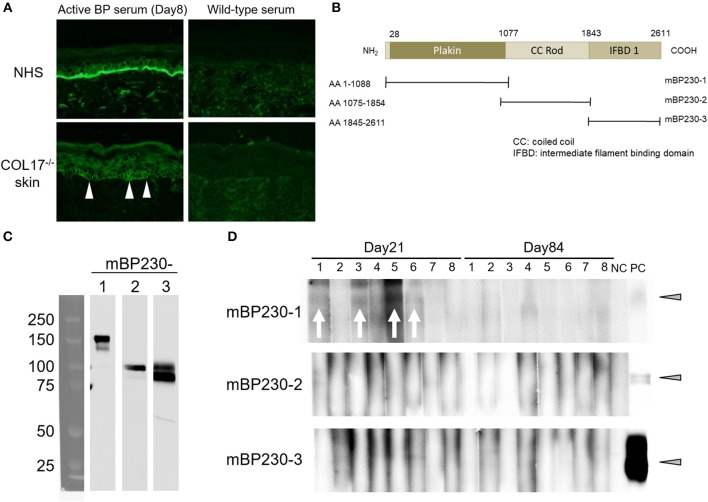
Antibodies to murine BP230 are detected in a portion of the active BP model mice. **(A)** Representative images of IgG deposition in indirect IF study using normal human skin (NHS) and murine COL17^−/−^ mouse skin as substrates and 1:20 diluted sera from an active BP model at day 35 and from wild-type mice as primary antibodies. Arrowheads indicate IgG deposition around the basal keratinocytes. **(B)** Schematic of murine BP230 and recombinant proteins. NH_2_, N-terminus; COOH, C-terminus; AA, amino acid; mBP230, murine BP230. **(C)** The sizes of recombinant proteins were confirmed by immunoblotting using anti-His-tag antibody. **(D)** Reactivity of sera from the active BP model (*n* = 8, days 21 and 84) to fragments of murine BP230 was measured by immunoblotting. NC, negative control (wild-type serum); PC, positive control (anti-His-tag antibody). Arrowheads indicate a positive level. Arrows indicate a positive band.

Next, we examined whether intermolecular epitope spreading occurs in an active BP model. First, we performed indirect IF analysis using sera from an active BP model and NHS containing human COL17, and COL17-deficient mouse skin lacking both murine and human COL17. As expected, sera from the active BP model strongly reacted to the DEJ of NHS; furthermore, they showed weak reactivity around the basal keratinocytes in COL17-deficient mouse skin (Figure 6A), suggesting the presence of antibodies to the antigens on basal keratinocytes other than COL17. Then, we focused on BP230, another major autoantigen of BP, and examined the presence of antibodies to murine BP230 by using recombinant proteins. Recombinant murine BP230 proteins were prepared as 3 fragments; the N-terminus domain (referred to as BP230-1), the rod domain (BP230-2) and the C-terminus domain (BP230-3) (37) (Figure 6B). The sizes were confirmed by Western blotting using anti-His-tag antibody (Figure 6C). We examined sera from skin-grafted wild-type mice at day 35 (*n* = 8), and they showed no reactivity (data not shown). We then examined sera from the active BP model at days 8, 21 and 84 (*n* = 8). No reactivity was observed in sera at day 8 (data not shown) nor at day 84 (Figure 6D), but interestingly, 4 sera at day 21 weakly reacted to BP230-1 (Figure 6D). No reactivity was observed on BP230-2 or BP230-3 fragments. There was no correlation between the appearance of antibodies to BP230-1 and disease severity (data not shown). Thus, the humoral immune response that started from COL17 extended to BP230 *in vivo*.

## Discussion

**Figure 7:**
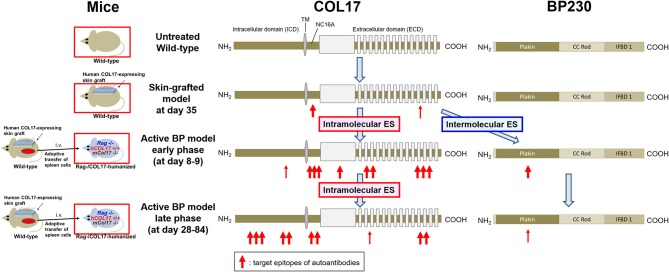
Epitope spreading in the skin-grafted mouse model and in the active BP model. A schematic of COL17 and BP230. In the skin-grafted mouse model, antibodies mainly react to the NC16A domain and weakly react to ECD epitopes of COL17. After the adoptive transfer of immunized spleen cells, intramolecular epitope spreading to ECD epitopes and to the outer portion of the ICD as well as intermolecular epitope spreading to BP230 occur, followed by intramolecular epitope spreading to the inner portion of the ICD. ES: epitope spreading.

This study has shown that intramolecular epitope spreading within COL17 and intermolecular epitope spreading from COL17 to BP230 occurs in experimental BP mouse models (Figure 7). Although the titer of antibodies to the NC16A domain drops rapidly (Figure 1E), the active BP model maintains a similar clinical disease severity for a long period (Figure 1C) in association with the clear deposition of IgG at the DEJ of lesional skin, even 35 days after the adoptive transfer of spleen cells as we previously reported (43). We had also noticed the discrepancy in time-course between the titer of antibodies to the DEJ as measured by indirect IF and that to the NC16A domain as measured by ELISA. In this study, we showed that this discrepancy is due to the delayed increase of antibodies to ICD epitopes of COL17. This is consistent with a previous study using a human COL17-expressing skin-grafted mouse model (33). In that study, antibodies to ICD epitopes emerged mostly later than 40 days after skin grafting, which is also consistent with our results in which antibodies to ICD-149 were not detected at day 35 in skin-grafted wild-type mice (Figure 3B). As reasons for the delayed increase of antibodies to ICD epitopes in the active BP model, we consider two possibilities. First, intramolecular epitope spreading from the NC16A domain to ICD epitopes occurs shortly after adoptive transfer. Second, ICD epitope-reactive T and B cells were already activated in skin-grafted wild-type mice as a result of intramolecular epitope spreading, and when these cells were transferred into *Ra*g^−/−^/COL17-humanized recipients, they encountered ICD antigens probably a little bit later than ECD antigens *in vivo*, resulting in the delayed peak of antibodies to ICD epitopes.

Unexpectedly, wild-type mice immunized with NC16A-R7 produced antibodies to ICD-149 (Figure 5B). We speculate that NC16A-R7-reactive T and B cells also recognized a corresponding site of murine COL17, which induced intramolecular epitope spreading to other epitopes of murine COL17 in wild-type mice. Although the homology of the amino acid sequence of NC17A-R7 (human COL17) and the corresponding site of murine COL17 is 54.1%, that of ICD-149 (human COL17) and the corresponding site of murine COL17 is 95.8%. The positive reactivity of NC16A-R7-immunized sera at day 35 to mouse skin shown in Figure 5C supports our hypothesis. Thus, intramolecular epitope spreading from the NC16A domain to ICD epitopes occurs in NC16A-immunized wild-type mice.

Two major mechanisms are considered to be behind the epitope spreading phenomenon: an “independent” or “dependent” of a physical association of antigens (30, 44). The former involves the development of secondary epitopes due to the release of antigens or the disclosure of parts of antigens during a chronic inflammatory or autoimmune response. The latter is independent of inflammatory processes. T cells specific for one epitope of an antigen can activate B cells that are specific for other antigens of the same multi-antigen complex, resulting in the generation of autoantibodies to antigens that are not initially targeted by the immune response (45). In this study, we demonstrated epitope spreading from the NC16A domain to other epitopes of COL17 in NC16A-R7-immunized wild-type mice, even though the mice developed no skin changes. This suggests that intramolecular epitope spreading within COL17 can occur under a mechanism that is “dependent” on a physical association of antigens.

We can estimate the timing of COL17-reactive T-cell activation more easily in the active BP mouse model than in the skin-grafted BP model, because in the former model, it starts soon after the adoptive transfer of spleen cells. By utilizing this advantage, we previously demonstrated that the activation of anti-human COL17 NC16A IgG-producing B cells via CD40–CD40L interaction is completed within 5 days after the transfer of spleen cells (41). The study also showed that antibodies to the DEJ were restored long after the single administration of antibodies to CD40L at day 0 and that the restored antibodies do not react to the NC16A domain. The results in Figure 4 suggest that the restored antibodies are mainly specific for ICD epitopes of COL17 and that the interaction between T and B cells regarding ICD epitopes may arise slightly bit later than that to the NC16A domain after the adoptive transfer of spleen cells. We previously reported that the blockade of CD40L at day 13, 16, and 19 after the adoptive transfer did not suppress antibodies to the NC16A domain (examined by ELISA), nor did it suppress antibodies to the DEJ (examined by indirect IF) (41). This strongly suggests that the delayed blockade of CD40-CD40L interaction does not suppress the production of antibodies to ICD epitopes and that the CD40-CD40L interaction between T and B cells regarding ICD epitopes was completed within 13 days after the adoptive transfer. The reason for the discrepancy between the results for NC16A-R7 and those for other ECDs in Figure 4 is unclear. As mentioned above, the reactivity of antibodies to NC16A-R7 is far stronger than those to ECD-917 and ECD-1330 in the untreated active BP model. This would be due to the high antigenicity of the NC16A domain of COL17 as reported previously (36, 46). It is commonly known that CD40L is transiently expressed on the surface of activated CD4^+^ T cells (47). Given the above, we consider that a treatment of CD40L-blocking antibody preferentially affects the highly activated T cells reacting to the NC16A domain rather than those reacting to the other ECD epitopes. Further investigations are required to confirm this.

This study also showed intermolecular epitope spreading from COL17 to BP230 in an active BP model. As mentioned above, intermolecular epitope spreading commonly occurs among different antigens of a single molecular complex or among antigens that colocalize to the same site (30). Both COL17 and BP230 are hemidesmosomal proteins, and they directly interact with each other at their N-termini (48). Di Zenzo et al. examined IgG reactivity to COL17 and BP230 during the course of the disease in 35 BP cases and detected epitope spreading in 17 cases (49%). Notably, 3 of those 17 cases showed intermolecular epitope spreading from COL17 to BP230, but the reactivity to BP230 never preceded that to COL17 (34). We demonstrated the reactivity of active BP mouse sera to murine BP230, but it was very weak, as shown in Figure 6D. This weak reactivity may be due to the strong self-tolerance in the wild-type mouse, a source of transferred spleen cells in the active BP model, because murine BP230 is originally expressed in wild-type mice. This hypothesis is supported by our recent finding that regulatory T cells, a main player in peripheral tolerance, play a crucial role in maintaining self-tolerance to murine BP230 in mice (37).

There are several limitations to this study. First, the synthesized peptides that were used for the ELISA account for only a small portion of human COL17. The titers of antibodies to ICD-1 (recombinant protein of 204 amino acids) were higher at day 56 than at day 8, as shown in Figure 2C, whereas the titers of antibodies to ICD-149 (synthesized peptides of 24 amino acids) were higher at day 8 than at day 56, as shown in Figure 3D. These results suggest that active BP mouse sera contain antibodies to ICD-1 epitopes (Met^1^ to Ser^204^) other than ICD-149 (Ala^149^ to Ser^172^). Second, a correlation between disease severity and epitope spreading remains unclear. As mentioned above, previous studies demonstrated that the disease severities of BP are associated with the appearance of antibodies to ICD epitopes in both mouse models and human patients (33, 34). Meanwhile the appearance of antibodies to BP230-1 or BP230 was not related to the disease severity of the active BP model in this study, which is probably due to the low titers of those antibodies. Third, we have not examined intramolecular epitope spreading that starts from ICD or ECD epitopes other than the NC16A domain. We recently reported 3 cases of dipeptidyl peptidase-4 inhibitor-associated BP in which autoantibodies to the NC16A domain were initially undetectable but became positive during the course of BP (49). To investigate this phenomenon, we immunized wild-type mice with recombinant proteins of ICD or ECD shown in Figure 2A and adoptively transferred the spleen cells to *Ra*g^−/−^/COL17-humanized mice. However, the titers of antibodies to COL17 were very low in all the immunized mice and the recipient mice (data not shown). This is probably due to the lower antigenicity of those proteins compared to that of the NC16A domain. Fourth, we have not examined intermolecular epitope spreading to hemidesmosomal proteins such as plectin, integrin α6, integrin β4, or laminin 332, which were reported to interact directly with COL17 (50–54), although those molecules become targets of autoantibodies less frequently than COL17 and BP230 do. We should also confirm whether the interaction between human COL17 and murine hemidesmosomal proteins in COL17-humanized mice is normal. To overcome these limitations, further investigations are necessary.

In conclusion, the immune reaction to human COL17 that starts from ECD epitopes, especially those of the NC16A domain, was found to spread over time to ICD epitopes and to murine BP230 in an experimental BP model. The timing of the interaction between COL17-reactive T and B cells differs *in vivo*, and it is dependent on the target epitope. These novel findings elucidate certain details of epitope spreading in BP and give us a hint for new therapeutic strategies for BP that involve the regulation of this phenomenon.

## Ethics Statement

This study was carried out in accordance with the recommendations of the local ethics committee and the Institutional Review Board of Hokkaido University, with written informed consent obtained from all subjects in accordance with the Declaration of Helsinki. All animal procedures were conducted according to guidelines provided by the Hokkaido University Institutional Animal Care and Use Committee under an approved protocol.

## Author Contributions

HU and NY performed the experiments. KN, KM, HI, and WN provided materials. HU and HS designed the experiments. HU wrote the manuscript, and all the coauthors reviewed the manuscript and gave final approval of the submission.

### Conflict of Interest Statement

The authors declare that the research was conducted in the absence of any commercial or financial relationships that could be construed as a potential conflict of interest.

## Abbreviations

BP: bullous pemphigoid
COL17: type XVII collagen
NC16A: non-collagenous 16A
ICD: intracellular domain
ECD: extracellular domain
CD40L: CD40 ligand
IF: immunofluorescence
ELISA: enzyme-linked immunosorbent assay
NHS: normal human skin
skin-grafted wild-type mice: wild-type mice grafted with human COL17-expressing skin
