# Supplementary figures and images for: Immune Reaction to Type XVII Collagen Induces Intramolecular and Intermolecular Epitope Spreading in Experimental Bullous Pemphigoid Models
Source: Front Immunol. 2019 Jun 19;10:1410. doi: 10.3389/fimmu.2019.01410 (PMC6593113; doi:10.3389/fimmu.2019.01410)

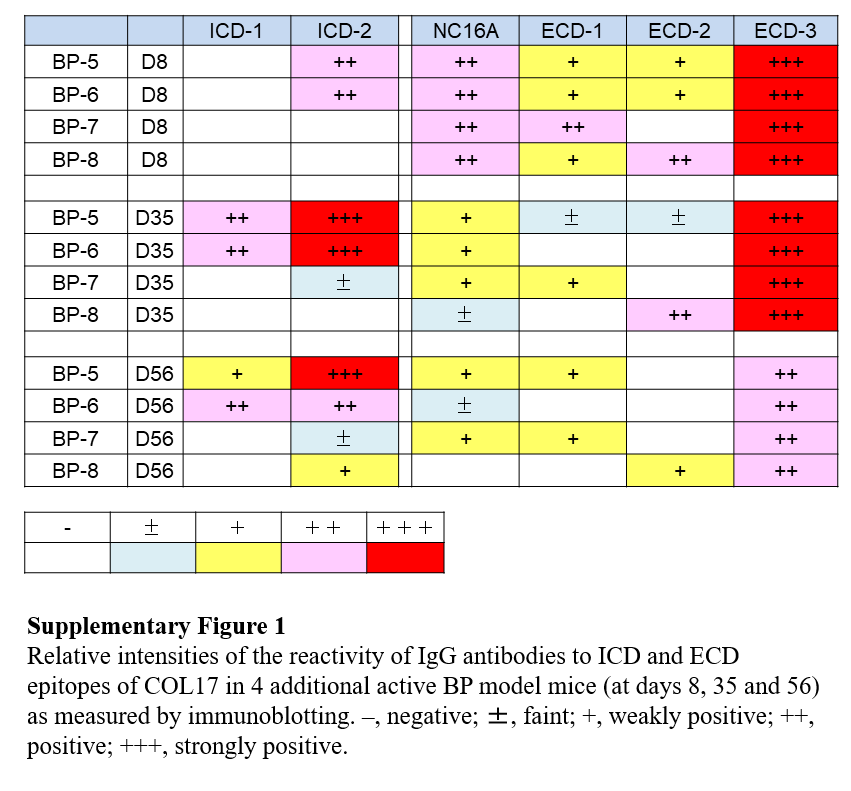

Supplement: Supplementary file 1 [file Image_1.TIF]
